# Phosphorylated IGFBP-1 as a non-invasive predictor of liver fat in NAFLD

**DOI:** 10.1038/srep24740

**Published:** 2016-04-19

**Authors:** Elina M. Petäjä, You Zhou, Marika Havana, Antti Hakkarainen, Nina Lundbom, Jarkko Ihalainen, Hannele Yki-Järvinen

**Affiliations:** 1 Minerva Foundation Institute for Medical Research, Helsinki, Finland; 2 Department of Medicine, University of Helsinki and Helsinki University Central Hospital, Helsinki, Finland; 3 Systems Immunity University Research Institute and Division of Infection and Immunity, School of Medicine, Cardiff University, Cardiff, United Kingdom; 4 United Medix Laboratories, Espoo, Finland; 5 HUS Medical Imaging Center, Radiology, University of Helsinki and Helsinki University Central Hospital, Helsinki, Finland

## Abstract

Insulin-like growth factor binding protein 1 (IGFBP-1) is a potentially interesting marker for liver fat in NAFLD as it is exclusively produced by the liver, and insulin is its main regulator. We determined whether measurement of fasting serum phosphorylated IGFBP-1 (fS-pIGFBP-1) helps to predict liver fat compared to routinely available clinical parameters and *PNPLA3* genotype at rs738409. Liver fat content (proton magnetic resonance spectroscopy) was measured in 378 subjects (62% women, age 43 [30–54] years, BMI 32.7 [28.1–39.7] kg/m^2^, 46% with NAFLD). Subjects were randomized to discovery and validation groups, which were matched for clinical and biochemical parameters and *PNPLA3* genotype. Multiple linear regression and Random Forest modeling were used to identify predictors of liver fat. The final model, % Liver Fat Equation’, included age, fS-pIGFBP-1, S-ALT, waist-to-hip ratio, fP-Glucose and fS-Insulin (adjusted R^2^ = 0.44 in the discovery group, 0.49 in the validation group, 0.47 in all subjects). The model was significantly better than a model without fS-pIGFBP-1 or S-ALT or S-AST alone. Random Forest modeling identified fS-p-IGFBP-1 as one of the top five predictors of liver fat (adjusted R^2^ = 0.39). Therefore, measurement of fS-pIGFBP-1 may help in non-invasive prediction of liver fat content.

NAFLD is closely associated with the metabolic syndrome (MetS) and predicts T2D independent of obesity^1^. Simple steatosis has recently been show to progress to non-alcoholic steatohepatitis (NASH) and clinically significant fibrosis^2^. Regarding diagnosis of NAFLD, a recent US Practice Guideline^3^ stated: “As liver biochemistries can be within normal ranges in patients with NAFLD, they may not be sufficiently sensitive to serve as screening tests… screening for NAFLD in adults attending primary care clinics or high-risk groups attending diabetes or obesity clinics is not advised at this time due to uncertainties surrounding diagnostic tests”. There is thus a need to develop such tests for NAFLD.

Insulin-like growth factor binding protein-1 (IGFBP-1) is one of six IGFBPs, which bind to and regulate bioavailability of insulin-like growth factor-1 (IGF-1)^4^. The liver exclusively produces IGFBP-1 in non-pregnant adults^5^. Insulin acutely decreases serum IGFBP-1 concentrations and is its major regulator *in vivo* in humans^6^. In addition to insulin, insulin sensitivity regulates serum IGFBP-1 concentrations^7^. fS-IGFBP-1 is lower in subjects with hepatic insulin resistance (IR) and high liver fat content than in subjects with preserved hepatic insulin sensitivity and low liver fat content^7^.

In NAFLD, fS-IGFBP-1 has been shown to be decreased in studies involving 142 Japanese subjects^8^, 48 Italian women^9^, and 49 African American and 77 Latino adolescents^10^. These studies did not compare models measuring IGFBP-1 alone or in combination with routinely available parameters associated with NAFLD such as age, gender, measures of obesity, glucose, insulin, lipids, liver enzymes to models without IGFBP-1.

Human hepatoma cells produce predominantly phosphorylated IGFBP-1 (pIGFBP-1)^11^. The majority of circulating IGFBP-1 is in a phosphorylated form^12^, which has the highest affinity for IGF-I^11^. These pIGFBP-1 assays utilize the antibodies and kits developed by one laboratory^13^,^14^ and thus avoid problems of standardization between laboratories unlike for measurement of e.g. insulin concentrations^15^. The concentration of phosphorylated but not other forms of IGFBP-1 associates with macrovascular complications in patients with T2D^16^, and pIGFBP-1 correlates better with cardiovascular risk factors than lesser-phosphorylated IGFBP-1^17^. These data provide a rationale for measuring specifically pIGFBP-1 rather than IGFBP-1 as a marker of liver fat content and associated metabolic abnormalities.

There are no data on the relationship between pIGFBP-1 and liver fat content or comparing pIGFBP-1 to routinely available markers in prediction of liver fat content. Furthermore, a common (30% to 50% of all subjects) variant in the patatin-like phospholipase domain-containing protein 3 (*PNPLA3)* gene at rs738409 (encoding I148M) increases liver fat content and risk of NASH but is not associated with features of IR^1^. Studies developing tools for non-invasive diagnosis NAFLD might thus benefit of genotyping for this gene variant. Previous studies have not considered this gene variant and have been performed in relatively small cohorts (48 to 142 subjects^8^,^9^,^10^). In the present study, we determined whether measurement of fS-pIGFBP-1 concentrations helps in non-invasive prediction of liver fat content, when features known to be associated with liver fat content^1^ such as age, gender, liver function tests, measures of obesity, and the *PNPLA3* genotype at rs738409 are taken into account. To this end, we measured these parameters, fS-pIGFBP-1 and liver fat content (proton magnetic resonance spectroscopy [^1^H-MRS]) in 378 subjects.

## Results

### Subject characteristics

Characteristics of the study subjects are shown in Table 1. Of all subjects, 46% had NAFLD. The discovery (n = 252) and validation (n = 126) groups were comparable with respect to clinical and biochemical parameters and *PNPLA3* genotype at rs739409 (Table 1).

### Univariate analysis

In univariate analysis in the discovery group, liver fat content was significantly inversely correlated with fS-pIGFBP-1 concentrations and significantly positively correlated with age, male gender, body mass index (BMI), waist circumference, increased liver enzyme, triglyceride, insulin and glucose concentrations, and the *PNPLA3* genotype at rs738409 (Table 2). The correlation coefficient between fS-Insulin and fS-pIGFBP-1 in all subjects was −0.51, *P* < 0.0001.

The variables were divided into groups measuring the same biological phenomenon such as measures of body composition or glycemia as shown in Table 2. The variables with best predictive value within each group in the discovery group, along with age, gender, fS-pIGFBP-1 and *PNPLA3* genotype at rs738409, were used in development of an equation for prediction of liver fat and in Random Forest analyses.

### Prediction of liver fat

**Figure 1:**
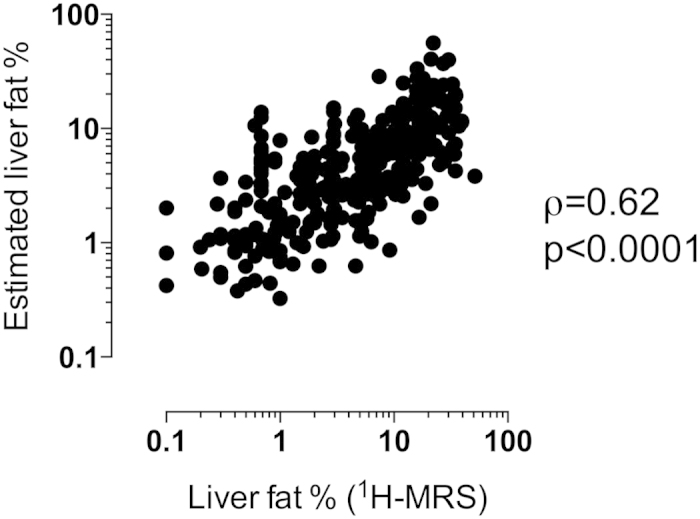
Spearman correlation between liver fat content measured using proton magnetic resonance spectroscopy (^1^H-MRS) and liver fat content estimated with the ‘% Liver fat equation’, ρ = 0.62 (95% CI 0.55–0.68), *P* < 0.0001.

The variables that had the best predictive value in the discovery group in univariate analysis were entered in multivariate linear regression analysis to create an equation for prediction of liver fat. The significant variables and their possible interactions were examined. The final variables for multiple linear regression analysis were derived using backward stepwise regression method based on Akaike Information Criteria (AIC). These variables were age, fS-pIGFBP-1, an interaction term (age times fS-pIGFBP-1), fS-alanine aminotransferase (ALT), waist-to-hip ratio, fasting plasma (fP)-Glucose and fS-Insulin. The final multiple linear regression model (‘% Liver fat equation’) in the discovery group (adjusted R^2^ = 0.44, *P* < 0.0001) is shown in Table 3. For calculation, Supplementary Table 1 can be used. The adjusted R^2^ was 0.49 in the validation group and 0.47 in all subjects. The adjusted R^2^ was 0.44 in all subjects, if fS-Insulin was omitted from the model (*P* < 0.0001 vs. the best model) and 0.46 if fS-pIGFBP-1 was omitted (*P* < 0.05 vs. the best model). The ‘% Liver fat equation’ also predicted liver fat significantly better than liver enzymes alone: aspartate aminotransferase (AST) only (adjusted R^2^ = 0.15), ALT only (adjusted R^2^ = 0.25), or both (adjusted R^2^ = 0.25, *P* < 0.0001 for all comparisons). The correlation coefficient between predicted liver fat content using ‘% Liver fat equation’ and liver fat measured using ^1^H-MRS was *ρ* = 0.62, *P* < 0.0001 (Fig. 1). The area under the receiver operator characteristic (AUROC) to predict NAFLD by ‘%Liver fat equation’ was 0.84 (0.80–0.88) which was significantly greater than that predicted by the Fatty Liver Index^18^ (0.72 [0.67–0.77], *p* < 0.0001) or the Hepatic Steatosis Index^19^ (0.62 [0.57–0.68], *p *< 0.0001).

**Figure 2:**
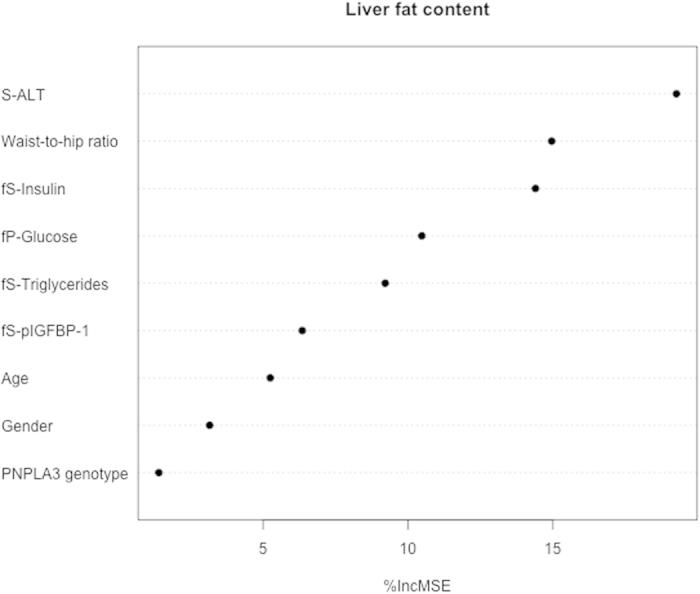
Random Forest model for prediction of liver fat content (%). Predictors were ranked by the importance score based on the percent increase in mean square error (%IncMSE), which measures the importance of a given variable in predicting liver fat content.

The best predictors in univariate analysis within each group were also subjected to Random Forest modeling for prediction of liver fat (Fig. 2). This approach identified S-ALT, waist-to-hip ratio, fS-insulin, fS-triglycerides and fS-pIGFBP-1 as the top five variables explaining variation in liver fat content. The adjusted R^2^ was 0.39 in all subjects.

## Discussion

We determined whether measurement of fS-pIGFBP-1 might help in the prediction of liver fat content in the face of other correlates of liver fat. The final model predicting liver fat included age, fS-pIGFBP-1, an interaction term (age times fS-pIGFBP-1), S-ALT, waist-to-hip ratio, fP-Glucose and fS-Insulin. The present data are novel in that we i) measured pIGFBP-1 rather than IGFBP-1, ii) did not study pIGFBP-1 in isolation but rather in combination with other markers of liver fat content including for the first time *PNPLA3* genotype. The present dataset is also the hitherto largest in which IGFBP-1 or pIGFBP-1 and liver fat content have been quantitated.

Early studies measuring IGFBP-1 did not specify to what extent the assay measured phosphorylated forms of IGFBP-1^8^,^9^,^10^,^20^,^21^. The median fS-pIGFBP-1 in the present study was 58 μg/l. This is in line with previously reported pIGFBP-1 concentrations ranging from 29 to 100^12^,^16^,^17^, which are markedly higher than concentrations of lesser-phosphorylated IGFPB-1 that range from 4 to 12 μg/l^16^,^17^. The phosphorylation status of IGFBP-1 alters its antigenicity^22^. Therefore immunoassays may grossly underestimate changes in IGFBP-1 concentrations^23^. In keeping with this, previous RIAs yielded mean fS-IGFBP-1 concentrations ranging from 16 to 20 μg/l^7^,^24^,^25^ and detected only a fraction of total IGFBP-1. Consistent with these data, in the subset of 23 subjects in the present study where we measured both IGFBP-1 using RIA and pIGFBP-1 using immunoenzymometric assay (IEMA), the mean concentration of fS-IGFBP-1 measured using RIA (18 μg/l) was much lower than that of fS-pIGFBP-1 measured using IEMA (58 μg/l).

The inverse relationship between liver fat content and pIGFBP-1 is consistent with previous data in diverse groups measuring IGFBP-1 using RIA or an immunoradiometric assay^7^,^8^,^9^,^10^. In the studies by Savastano *et al*. in 48 subjects^9^ and Kotronen *et al*. in 113 subjects^7^, the correlation coefficients between fS-IGFBP-1 and hepatic steatosis score (ultrasound) or liver fat (^1^H-MRS) were in both studies −0.38 (*P* < 0.01 or less). In the present study, the correlation coefficient between fS-pIGFBP-1 and liver fat (^1^H-MRS) in 378 subjects was −0.29 (*P* < 0.0001) and thus statistically comparable to the previous data in smaller groups of subjects (*P* = 0.5 for *r* = −0.29 in 378 subjects vs. *r* = −0.38 in 48 subjects^9^ and *P* = 0.3 for *r* = −0.29 in 378 subjects vs. *r* = −0.38 in 113 subjects^7^).

Multiple causes (measures of obesity, aging) and consequences (hypertriglyceridemia, hyperinsulinemia, hyperglycemia, increased liver enzymes) of IR are known to be significantly associated with increased liver fat^1^. This was also true in the present study and emphasizes the need to consider several parameters rather than one parameter in isolation when developing tools for non-invasive prediction of liver fat (Table 2). In addition, carriers of the I148M variant in the *PNPLA3* gene at rs738409 have increased liver fat and liver enzyme concentrations but no features of IR^26^. We found the *PNPLA3* I148M gene variant to be significantly associated with liver fat in univariate (Table 2) but not in multiple linear regression (Table 3) analyses. This could be because the *PNPLA3* gene variant signals its influence via ALT, which remained a significant independent predictor in multiple linear regression analysis (Table 3). In addition to these established markers, fS-pIGFBP-1 was significantly associated with liver fat content in both univariate and multiple linear analyses.

In line with the significant interaction between age and fS-pIGFBP-1, IGFBP-1 has been shown to correlate with age independent of BMI^27^. Aging also associates with decreased suppression of IGFBP-1 by insulin^27^. Regarding mechanisms underlying the observed inverse relationship between fS-Insulin and fS-pIGFBP-1, the subjects with increased liver fat content also were hyperinsulinemic (Table 2). Thus the relationship could reflect insulin inhibition of production of IGFBP-1 in the liver^5^. Hepatic IR could also influence the slope of the relationship between fS-Insulin and fS-IGFBP-1. A fixed increment in serum insulin suppresses serum IGFBP-1 less in insulin-resistant than -sensitive subjects^7^. Of these two factors, i.e., insulin per se and hepatic insulin sensitivity, insulin may be the most important regulator of fS-IGFBP-1, as type 1 diabetic patients who lack the portal-peripheral insulin gradient have markedly higher fS-IGFBP-1 concentrations than matched non-diabetic subjects despite enhanced hepatic insulin sensitivity^28^,^29^.

We acknowledge limitations in our study. First, the study was cross-sectional and hence fails to prove cause and effect. Second, even when a multitude of factors known to be either causes or consequences of liver fat content were considered, a large proportion of the variation in liver fat remains unexplained. Direct measurement of liver fat content by ultrasound therefore would seem to be a more attractive tool, as it is widely available. This method has, however, the limitation that it lacks sensitivity in subjects with low liver fat content^30^ and accuracy in obese subjects^31^. ^*1*^H-MRS is considered to be the most accurate non-invasive method for assessing hepatic steatosis^32^ and it also does not expose to radiation but it is expensive and requires MRI and hence is not widely available. Compared to measurement of e.g. liver enzymes alone, the ‘%Liver fat equation’ was much better in capturing information of liver fat. The AUROC of the ‘%Liver fat equation’ (0.84) was significantly better than that of the Fatty liver Index (0.72) or the Hepatic Steatosis Index (0.62).

The variables included in equations predicting liver fat should be standardized to enable comparison between different laboratories and centers. Although fS-Insulin is perhaps the most popular laboratory test to assess insulin sensitivity, assay procedures are highly variable and measure various forms of insulin using divergent procedures^15^. In this context it is noteworthy that measurement of pIGFBP-1 for predicting pre-term delivery^13^,^14^ and lesser-phosphorylated IGFBP-1 for diagnosis of premature rupture of fetal membranes^33^ produced by a single manufacturer have become a worldwide standard. We thus conclude that measurement of fS-pIGFPB1 independently contributes to prediction of liver fat even when the known associates are considered and may thus be helpful in non-invasive estimation of liver fat content.

## Materials and Methods

### Subjects

The subjects (n = 378) were recruited for metabolic studies^34^ by newspaper advertisements, by contacting occupational health services, or amongst subjects referred to the Department of Gastroenterology because of chronically elevated serum transaminase concentrations using the following inclusion criteria: (i) age 18 to 75 years; (ii) no known acute or chronic disease except obesity or T2D based on medical history, physical examination, standard laboratory tests and electrocardiogram; (iii) alcohol consumption of less that 20 g per day. Study physicians assessed alcohol intake by using the same questionnaire addressing the quantity of different alcoholic drinks consumed during an average week. Exclusion criteria included pregnancy, serologic evidence of hepatitis B or C, autoimmune hepatitis, clinical signs or symptoms of inborn errors of metabolism, or a history of use of toxins or drugs associated with liver steatosis, antihypertensives possibly influencing glucose metabolism or thiazolidinediones. The study protocol was approved by the ethics committee of the Helsinki University Central Hospital and was carried out in accordance with the Declaration of Helsinki. Each participant provided written informed consent.

### Metabolic study

The subjects were studied after an overnight fast. Body composition was measured as detailed below. Blood was withdrawn for measurement of plasma glucose concentration, and serum total, HDL and low-density lipoprotein (LDL) cholesterol, triglyceride, glycosylated hemoglobin A_1c_ (HbA_1c_), insulin, C-peptide, pIGFBP-1, ALT, and AST concentrations. Blood samples were also taken for genotyping *PNPLA3* at rs738409^35^.

### Measurement of body composition

Waist circumference was measured midway between spina iliaca superior and the lower rib margin, and hip circumference at the level of the greater trochanters. Body weight was recorded to the nearest 0.1 kg using a calibrated digital scale (Soehnle, Monilaite-Dayton, Finland) with subject barefoot and wearing light indoor clothing. Height was recorded to the nearest 0.5cm using a non-stretchable tape. BMI was defined as [weight (kg)/(height (m))^2^]. Body fat percentage was determined using a bioelectric impedance analysis (BioElectrical Impedance Analyzer System model #BIA-101A; RJL Systems, Detroit, MI).

### Liver fat content measured using ^1^H-MRS

Liver fat was measured using ^1^H-MRS^34^. In ^1^H-MRS studies, the intensity differences arising from various acquisition parameters and localization techniques were normalized as previously described and liver fat content was expressed as mass fraction^34^. We have previously validated this measurement against histologically determined liver fat content^36^. NAFLD was defined as in the Dallas Heart Study (liver fat ≥5.56% by ^1^H-MRS)^37^.

### Analytical procedures

Plasma glucose was measured using a hexokinase method on an autoanalyser (Roche Diagnostics Hitachi 917, Hitachi Ltd., Tokyo, Japan). Serum insulin and C-peptide concentrations were measured using time-resolved fluoroimmunoassay with respective Auto-DELFIA kits (Wallac, Turku, Finland). Serum HbA_1c_ was measured using high-pressure liquid chromatography using the fully automated analyzer system (Bio-Rad, Richmond, CA). Serum triglyceride, total and HDL cholesterol concentrations were measured using the enzymatic kits from Roche Diagnostics using an autoanalyzer (Roche Diagnostics Hitachi, Hitachi Ltd., Tokyo, Japan). Serum LDL cholesterol concentrations were calculated using the Friedewald formula^38^. Serum ALT and AST activities were determined as recommended by the European Committee for Clinical Laboratory Standards using the Roche Diagnostics Hitachi 917.

Serum pIGFBP-1 concentrations were determined with an IEMA with a monoclonal antibody as the detecting antibody (Medix Biochemica, Kauniainen, Finland) according to the manufacturer’s instructions, as described before^39^. The assay uses a monoclonal antibody specific to human pIGFBP-1, which is immobilized on microwell plates, and a monoclonal antibody specific to IGFBP-1, which is conjugated with horse-radish peroxidase. The intra- and interassay coefficients of variation were 2.7% to 7.8% and 3.9% to 10%, respectively. Each sample was assayed as a duplicate and the mean value was used. The detection limit the assay was 0.3 *μ*g/l and the measuring range 1 to 200 *μ*g/l. No cross-reactivity between other IGFBPs was seen. All sera were analyzed after storage at −80 C until analysis. The present series included 23 samples, which had been collected 5 years ago and in which fS-IGFBP-1 had been measured using a radioimmunoassay (RIA)^7^. These samples were re-assayed with the IEMA detecting fS-pIGFBP-1. The mean concentrations were 18 ± 2 *μ*g/l with the RIA and 57 ± 8 *μ*g/l with the IEMA (*P* < 0.001). The correlation coefficient between the two measurements was 0.64, *P* < 0.001 (Suppl. Fig. 1). In these samples, the correlation coefficient between liver fat and pIGFBP-1 was −0.45 (*P* = 0.029) and IGFBP-1 −0.45 (*P* = 0.033).

### Statistical analyses

The subjects were randomly divided using bootstrap randomization into discovery (2/3 of the subjects) and validation (1/3 of the subjects) groups. All subjects were used as the second validation group. Normality of distribution of continuous variables was tested using Kolmogorov-Smirnov test. The Mann-Whitney U and Chi-square tests were used to compare the validation and discovery groups. Spearman’s rank correlation coefficient was used for univariate analysis. Liver fat content and circulating parameters were used as continuous variables and non-normally distributed data after logarithmic (base 10) transformation.

We used linear regression and Random Forest prediction models to estimate the liver fat content. For both models, we entered variables that significantly correlated with liver fat in univariate analyses in the discovery group. To avoid multi-collinearity, we entered one variable from a group of variables reflecting the same biological phenomenon (body composition, liver enzymes, glycemia, insulinemia, and lipids) as shown in Results in Table 2. The final variables for multiple linear regression analysis were derived using backward stepwise regression method based on AIC. We applied multiple linear regression analyses to create an equation to estimate liver fat content and evaluated the model using adjusted coefficient of determination (R^2^). Predictive models were compared using the F-test based on the residual sum of squares adjusted for the total number of variables in each model. To compare the accuracy of the equation with the Fatty Liver Index^18^ and the Hepatic Steatosis Index^19^, we used their respective reference values and for the created equation the 5.56% reference value^37^ as a cut-off for NAFLD and compared the AUROCs using the DeLong method^40^. In the Random Forest modeling, the optimal number of variables on each tree was defined based on the estimation of out-of-Bag error. By using the predictors described above, 500 regression trees were trained in the discovery group. The predictability of each variable was estimated by cross-validating its relationship with the outcome in the validation group and all subjects. A variable importance plot based on the importance score summarized the importance of each predictor. Correlation coefficients were compared statistically using the Fisher r-to-z transformation^41^.

We considered a *P*-value of less than 0.05 statistically significant. Using a sample size of 378 subjects, a power of 80% and a *P*-value of 0.05, a linear correlation coefficient of 0.144 or over can be detected. Calculations were made using R Project for Statistical Computing version 3.1.1 (www.r-project.org/, Vienna, Austria) and GraphPad Prism version 6.00 for Windows (GraphPad Software, San Diego, CA).

## Additional Information

**How to cite this article**: Petäjä, E. M. *et al*. Phosphorylated IGFBP-1 as a non-invasive predictor of liver fat in NAFLD. *Sci. Rep*. **6**, 24740; doi: 10.1038/srep24740 (2016).

## Supplementary Material

Supplementary Information

## Figures and Tables

**Table 1:** Characteristics of subjects.

	Discovery group(*n* = 252)	Validation group(*n* = 126)	All subjects(*n* = 378)
Age (*years*)	44 (31–55)	40 (28–53)	43 (30–54)
Gender (*women/men*)	152/100	81/45	233/145
fS-pIGFBP-1 (*μg/l*)	56 (33–109)	67 (28–96)	58 (32–106)
*PNPLA3* genotype (*I148II/IM/MM*)	56%/35%/10%	49%/42%/9%	52%/36%/9%
Liver fat%	5.0 (1.0–12.0)	4.7 (1.0–12.0)	4.9 (1.0–12.4)
NAFLD (*no/yes*)	134/118	70/56	204/174
S-ALT (*U/l*)	33 (21–51)	30 (20–53)	32 (21–51)
S-AST (*U/l*)	28 (23–40)	29 (22–40)	28 (23–40)
Weight (*kg*)	92.3 (79.9–118.2)	97.0 (81.5–114.7)	94.2 (80.9–115.2)
BMI (*kg/m^2^*)	32.6 (27.7–39.5)	33.0 (28.9–40.7)	32.7 (28.1–39.7)
Waist circumference (*cm*)	107 (95–122)	107 (97–122)	107 (96–122)
Waist-to-hip ratio	0.94 (0.80–1.01)	0.96 (0.89–1.03)	0.94 (0.88–1.02)
Body fat*%*	35 (26–42)	35 (29–40)	35 (27–41)
fP-Glucose (*mmol/l*)	5.8 (5.3–6.7)	5.7 (5.1–6.2)	5.7 (5.2–6.5)
HbA_1c_ (*%*)	5.8 (5.4–6.3)	5.7 (5.3–6.1)	5.7 (5.4–6.2)
fS-Insulin (*mU/l*)	9.0 (6.0–15.2)	9.7 (6.0–15.7)	9.3 (6.0–15.0)
fS-C-peptide (*nmol/l*)	0.90 (0.58–1.18)	0.92 (0.64–1.18)	0.94 (0.58–1.23)
fS-Triglycerides (*mmol/l)*	1.3 (1.0–1.9)	1.4 (1.0–1.8)	1.3 (1.0–1.9)
fS-HDL cholesterol (*mmol/l)*	1.3 (1.0–1.6)	1.2 (1.1–1.6)	1.3 (1.0–1.6)
fS-LDL cholesterol (*mmol/l)*	2.7 (2.1–3.3)	2.8 (2.2–3.4)	2.6 (2.1–3.4)

Data are shown as median (25–75%). All comparisons between the discovery and validation groups were non-significant (Mann Whitney U and Chi square tests, accordingly).

**Table 2:** Univariate analysis of correlates of liver fat% (^1^H-MRS) (Spearman *ρ*).

	Discovery group (*n* = 252)		Validation group (*n* = 126)		All subjects (*n* = 378)	
	Spearman ρ	*P*-value	Spearman ρ	*P*-value	Spearman ρ	*P*-value
Age (*years*)	0.25	<0.0001	0.087	ns	0.24	<0.0001
Gender (*women/men*)	0.14	0.02	0.32	0.003	0.14	0.007
fS-pIGFBP-1 (*μg/l*)	−0.21	0.0009	−0.41	<0.0001	−0.27	<0.0001
*PNPLA3* genotype (*I148M allele*)	0.16	0.01	0.092	ns	0.11	0.03
*Liver enzymes*						
S-ALT (*U/l*)	0.46	<0.0001	0.48	<0.0001	0.48	<0.0001
S-AST (*U/l*)	0.37	<0.0001	0.32	<0.0001	0.37	<0.0001
*Body composition*						
Weight (*kg*)	0.20	0.002	0.35	<0.0001	0.24	<0.0001
BMI (*kg/m^2^*)	0.17	0.005	0.39	<0.0001	0.24	<0.0001
Waist circumference (*cm*)	0.28	<0.0001	0.47	<0.0001	0.34	<0.0001
Waist-to-hip ratio	0.41	<0.0001	0.49	<0.0001	0.44	<0.0001
Body fat%	0.17	0.022	0.32	0.0009	0.19	0.0008
*Glycemia*						
fP-Glucose (*mmol/l*)	0.42	<0.0001	0.41	<0.0001	0.42	<0.0001
HbA_1c_ (*%*)	0.40	<0.0001	0.48	0.0002	0.43	<0.0001
*Measures of Insulinemia*						
fS-Insulin (*mU/l*)	0.46	<0.0001	0.56	<0.0001	0.49	<0.0001
fP-C-peptide (*nmol/l*)	0.32	0.0002	0.43	<0.0001	0.35	<0.0001
*Lipids*						
fS-Triglycerides (*mmol/l)*	0.40	<0.0001	0.41	<0.0001	0.40	<0.0001
fS-LDL cholesterol (*mmol/l)*	0.10	ns	0.16	ns	0.11	0.034
fS-HDL cholesterol (*mmol/l)*	−0.25	<0.0001	−0.36	0.042	−0.29	<0.0001

**Table 3:** Multiple linear regression analysis.

Liver fat (*log*, %) Adjusted R^2^ = 0.44, *P* < 0.0001	Betacoefficient	Standarderror	*P*-value
Age (*years, log*)	−2.707	1.376	0.05
fS-pIGFBP-1 (*μg/l, log*)	−2.635	1.177	0.026
fS-pIGFBP-1 (*log*) x Age (*log*)	1.644	0.735	0.026
S-ALT (*U/l, log*)	0.571	0.127	<0.0001
Waist-to-hip ratio *(log)*	2.813	0.957	0.0037
fP-Glucose (*mmol/l, log*)	1.064	0.350	0.0027
fS-Insulin (*mU/l, log*)	0.393	0.125	0.0019
Constant	0.960	0.735	<0.0001
