# Supplementary material for: Phosphorylated IGFBP-1 as a non-invasive predictor of liver fat in NAFLD
Source: Sci Rep. 2016 Apr 19;6:24740. doi: 10.1038/srep24740 (PMC4835723; doi:10.1038/srep24740)
Supplement: Supplementary Information [file srep24740-s1.doc]

**Phosphorylated IGFBP-1 as a non-invasive predictor of liver fat in NAFLD**

Elina M. Petäjä^1, 2*^, You Zhou^1^, Marika Havana^3^, Antti Hakkarainen^4^,Nina Lundbom^4^, Jarkko Ihalainen^3^, Hannele Yki-Järvinen^1, 2^

Supplementary material

1. Supplementary figure 1.
2. Suppelementary table 1.

**Supplementary figure 1.**


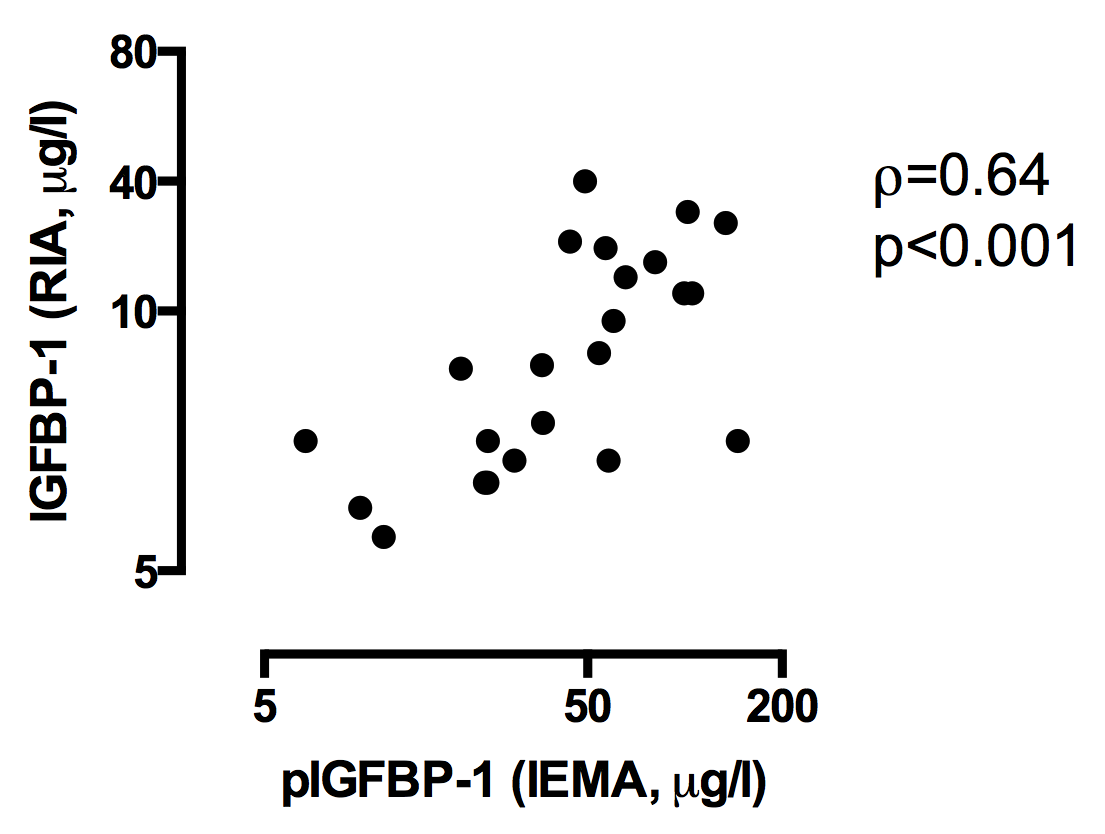


Spearman correlation between fS-IGFBP-1 measured by RIA and fS-pIGFBP-1 measured by IEMA in 23 subjects (0.64, *P*<0.001).

|  | **Supplementary table 1. ‘%Liver fat equation’ as an excel file** |  |  |  |  |
| --- | --- | --- | --- | --- | --- |
|  |  |  |  |  |  |
|  | Age |  | years |  |  |
|  | Waist-hip-ratio |  |  |  |  |
|  | Fasting serum ALT |  | U/l |  |  |
|  | Fasting serum insulin |  | mU/l |  |  |
|  | Fasting plasma glucose |  | mmol/l |  |  |
|  | Fasting serum pIGFBP-1 |  | µg/l |  |  |
|  | **Liver fat (%) =** |  |  |  |  |
|  | | | | | |
|  | | | | | |
|  | | | | | |

Liver fat (%)=100*10^(0.9559-2.707*log^_10_^(age^, *^years^*^)+0.5708*log^_10_^(fS-ALT^, *^U/l^*^)+2.813*log^_10_^(waist-to-hip ratio)+0.363*log^_10_^(fS-insulin^, *^mU/l^*^)+1.064*log^_10_^(fP-glucose^, *^mmol/l^*^)-2.6351*log^_10_^(fS- pIGFBP-1^, ^µ^*^g/l^*^)+1.6439*log^_10_^(age^, *^years^*^)*log^_10_^(fS-pIGFBP-1, µ^*^g/l^*))

The equation is provided in an excel file where the values can be submitted into and the calculator will estimate liver fat content accoring to the ’%Liver fat equation’
